# A logical analysis of null hypothesis significance testing using popular terminology

**DOI:** 10.1186/s12874-022-01696-5

**Published:** 2022-09-19

**Authors:** Richard McNulty

**Affiliations:** Emergency Department, Blacktown Mount Druitt Hospitals, Blacktown Rd, Blacktown, Sydney, NSW 2148 Australia

**Keywords:** Logic, Null hypothesis significance test, Hypothesis testing, Statistical inference, Statistical significance, Type I error, Type II error, Power

## Abstract

**Background:**

Null Hypothesis Significance Testing (NHST) has been well criticised over the years yet remains a pillar of statistical inference. Although NHST is well described in terms of statistical models, most textbooks for non-statisticians present the null and alternative hypotheses (*H*_0_ and *H*_A_, respectively) in terms of differences between groups such as (*μ*_1_ = *μ*_2_) and (*μ*_1_ ≠ *μ*_2_) and *H*_A_ is often stated to be the research hypothesis. Here we use propositional calculus to analyse the internal logic of NHST when couched in this popular terminology. The testable *H*_0_ is determined by analysing the scope and limits of the *P*-value and the test statistic’s probability distribution curve.

**Results:**

We propose a minimum axiom set NHST in which it is taken as axiomatic that *H*_0_ is rejected if *P*-value< *α*. Using the common scenario of the comparison of the means of two sample groups as an example, the testable *H*_0_ is {(*μ*_1_ = *μ*_2_) and [($\overline{x}$_1_ ≠ $\overline{x}$_2_) due to chance alone]}. The *H*_0_ and *H*_A_ pair should be exhaustive to avoid false dichotomies. This entails that *H*_A_ is ¬{(*μ*_1_ = *μ*_2_) and [($\overline{x}$_1_ ≠ $\overline{x}$_2_) due to chance alone]}, rather than the research hypothesis (*H*_T_). To see the relationship between *H*_A_ and *H*_T_, *H*_A_ can be rewritten as the disjunction *H*_A_: ({(*μ*_1_ = *μ*_2_) ∧ [($\overline{x}$_1_ ≠ $\overline{x}$_2_) not due to chance alone]} ∨ {(*μ*_1_ ≠ *μ*_2_) ∧ [$(\overline{x}$_1_ ≠ $\overline{x}$_2_) not due to (*μ*_1_ ≠ *μ*_2_) alone]} ∨ **{(*μ***_**1**_ **≠** ***μ***_**2**_**) ∧ [(**$\overline{\boldsymbol{x}}$_**1**_ **≠** $\overline{\boldsymbol{x}}$_**2**_**) due to (*μ***_**1**_ **≠** ***μ***_**2**_**) alone]}**). This reveals that *H*_T_ (the last disjunct in bold) is just one possibility within *H*_A_. It is only by adding premises to NHST that *H*_T_ or other conclusions can be reached.

**Conclusions:**

Using this popular terminology for NHST, analysis shows that the definitions of *H*_0_ and *H*_A_ differ from those found in textbooks. In this framework, achieving a statistically significant result only justifies the broad conclusion that the results are not due to chance alone, not that the research hypothesis is true. More transparency is needed concerning the premises added to NHST to rig particular conclusions such as *H*_T_. There are also ramifications for the interpretation of Type I and II errors, as well as power, which do not specifically refer to *H*_T_ as claimed by texts.

## Background

Null Hypothesis Significance Testing (NHST^1^) and the Confidence Interval (CI) or estimation method are the pillars of statistical inference [1–5]. NHST is perhaps the more common of the two for the analysis of research questions [6]. In NHST a null hypothesis (*H*_0_) is rejected in favour of an alternative hypothesis (*H*_A_) only if the *P*-value, *P* (observed data or more extreme│*H*_0_), falls below a pre-specified *α*-level. The latter is the maximum probability we are prepared to tolerate of erroneously rejecting *H*_0_. If the *P*-value is less than *α*, then this is called a statistically significant result and *H*_0_ can be rejected. Some familiarity with NHST will be assumed in this paper. NHST is a combination of two different statistical theories: R. A. Fisher’s *P*-value significance test, and the Neyman-Pearson technique of hypothesis testing. The two groups never intended to unite the theories, with well-known antagonisms existing between them [7]. However, NHST gained traction perhaps due to its appeal as a mechanical decision tool. Parallel to its popularity is the detailed, sharp criticism it has received from several quarters. Problems raised include: the misinterpretation of the *P*-value as *P*(*H*_0_│observed data) rather than *P* (observed data or more extreme│*H*_0_); the artificial dichotomous nature of statistical significance; and the conflation of statistical significance with clinical importance [8]. In fact, *P*-values have even been temporarily banned from some journals [9]. More recently, the correct level of statistical significance (*P*-value or *α* cut-off) has again been debated [10]. However, rather than cover old ground, we will here present a new logical analysis of a popular version of NHST presented in textbooks. NHST is perhaps best explained in terms of statistical models [11]. However, in most popular textbooks for non-statisticians, NHST is frequently presented in terms of the difference between population or sample groups and framed in reference to the research hypothesis. The need for an in-depth focus on the logic of NHST when couched in these terms can be seen from the following summary.

Starting with *H*_0_, there are various definitions offered. *H*_0_ is the hypothesis of no difference or association between groups [1, 5, 12–27]. Using population means (*μ*) as an example, this is *H*_0_: *μ*_1_ = *μ*_2_, meaning there is no difference in the population [2, 28–33]. In addition, there is the idea that *H*_0_ is the opposite/reverse/complement/negation of the test/experimental/study/research hypothesis [1, 3, 6, 25, 27, 28]. In clinical studies, this segues to the stronger claim that the absence of a difference is due to a lack of treatment effect [3, 5, 6, 13, 20, 21, 28, 31, 34–36]. In contrast to the idea of “no difference” is the anticipation that chance or random variation will produce a difference between the sample means [37]. Some texts unite the two ideas about the presence and absence of difference into one *H*_0_ which states there is no difference in the population and the difference in the sample groups is due to chance [2, 38–41]. Although a symbol exists for the mean of the sample group $(\overline{x})$, there was no example of this more complex version of *H*_0_ translated into symbols in any text sampled. In fact, some texts mention this more complex *H*_0_ only to quickly drop the idea and revert to *H*_0_: *μ*_1_ = *μ*_2_ anyway [27, 42].

Moving on to the definition of *H*_A_, we find similar themes phrased in a contrary fashion. *H*_A_ is the hypothesis that there is a difference or association between the groups [12, 13, 22, 23, 32]. Some specify that the groups are the populations such that *H*_A_: *μ*_1_ ≠ *μ*_2_ [2, 4, 24]. This type of difference is described as statistically significant [26] or real [2, 17, 18, 42, 43]. *H*_A_ is elsewhere proposed to be: the experimental/ research/study hypothesis [3, 5, 6, 28, 36, 43]; or the hypothesis that there is a treatment effect [1, 6, 20, 33, 34, 39]; or the contradictory or complementary hypothesis to *H*_0_ [14, 34, 35, 42]. There are attempts to unite claims about the population and sample groups, namely that the difference in the sample groups is due to the difference in the population [42]. Again, in the texts sampled, the latter hypothesis was never translated into symbols or further pursued.

Another area of disagreement, apart from the content of *H*_A_, is the strength of the conclusion when rejecting *H*_0_. Some claim we accept *H*_A_ as true [1, 5, 16, 20, 23] or real [18]. There are also softer versions that state *H*_A_ is just “supported” or is “probably true” [6, 19]. Alternatively, conclusions can be framed in terms of the test hypothesis being true [2, 15, 16, 20, 27, 29, 33–35, 43, 44], or more tentatively, that we gain confidence or support for the test hypothesis [6, 25, 28, 31, 41, 42]. More bewildering still are claims suggesting there are multiple other hypotheses or explanations! [1, 12, 16, 21, 34, 35, 40]

The interpretation of the phrase “statistically significant” [2, 5, 21, 34, 39, 40, 42], often abbreviated to just “significant” [21, 25, 27, 28, 30, 33–35], ranges from the claim that the data are not due to chance [24, 45] to the weaker claim that the data are unlikely to be due to chance [2, 18, 40].

In NHST, *H*_0_ and *H*_A_ are presented as a hypothesis pair. A commonly presented pair is *H*_0_: *μ*_1_ = *μ*_2_ and *H*_A_: *μ*_1_ ≠ *μ*_2_. This hypothesis pair is mutually exclusive and exhaustive which some texts explicitly state are desirable characteristics [1, 19, 46]. Elsewhere, however, *H*_0_ and *H*_A_ are frequently presented as a non-exhaustive, false dichotomy between the test hypothesis and the hypothesis that the results are due to chance [3, 6, 16, 18, 19, 24, 25, 27, 34, 38, 40, 41, 44].

From the above we see that this family of interpretations of NHST provides no consensus on many aspects. This poses a challenge to interpreting NHST when expressed in this fashion. From within the framework of this popular terminology, the purpose of the present paper is to

- 1/ define *H*_0_, *H*_A_, power and type I and II errors,
- 2/ define the minimum axiom set for NHST and
- 3/ make transparent which assumptions are needed to conclude the research hypothesis is true.

## Methods

Here we assume the common terminology of expressing NHST in terms of differences between populations or sample groups and in reference to the research hypothesis. The scope and limits of the *P*-value, the test statistic and its probability distribution curve (PDC) will be used to arbitrate on the correct form of *H*_0_ and *H*_A_ within this framework. Propositional calculus will be employed to analyse NHST. We also acknowledge multi-factorial hypotheses. For example, we can hypothesise that the difference between two sample groups is due to bias, chance or an intervention. These hypotheses are independent which entails that they can act in combination to produce the results. To disambiguate between single- or multi-factorial hypotheses, the term “alone” will be used to refer to the former. For example, “($\overline{x}$_1_ ≠ $\overline{x}$_2_) due to chance alone” means chance is the only factor involved in the sample group difference, as opposed to chance acting in concert with other factors to produce the results.

## Results

For consistent vocabulary throughout this paper, we will use as our example the common scenario of comparing the means of two sample groups. The appropriate test statistic for this is the *t*-statistic which has its relevant PDC. We will commence by stating the minimum axiom set needed for a NHST to function. To this end, we accept as axiomatic that if *P*(observed data or more extreme│*H*_0_) < *α*, then reject *H*_0_ and accept *H*_A_.

### The testable *H*_0_

In the introduction we saw that *H*_0_ had various definitions including *H*_0_: *μ*_1_ = *μ*_2_ or the “opposite” of the research hypothesis. Understandably, these are *H*_0_’s that we would like to test, but that does not guarantee that these candidates are testable. Here we propose a new approach: the decision concerning which is the correct *H*_0_ should be determined by the scope and limits of the actual technique that will be used to reject *H*_0_. In our example, the decision to reject *H*_0_ is based on the *P*-value of the *t*-statistic read off from its PDC. The PDC yields the probability of finding the observed *t*-statistic value (or more extreme) due to chance alone when there is no difference in the population means. In symbols, (something which never appeared in the texts mentioned in the introduction), the PDC gives us

$$P\left(\mathrm{observed}\ t-\mathrm{statistic}\ \mathrm{value}\ \mathrm{or}\ \mathrm{more}\ \mathrm{extreme}\vert \left\{\left({\mu}_1={\mu}_2\right)\ \mathrm{and}\ \left[\left({\overline{x}}_1\ne {\overline{x}}_2\right)\ \mathrm{due}\ \mathrm{to}\ \mathrm{chance}\ \mathrm{alone}\right]\right\}\right).$$

Given that the definition of the *P*-value is

$$P\left(\mathrm{observed}\ t-\mathrm{statistic}\ \mathrm{value}\ \mathrm{or}\ \mathrm{more}\ \mathrm{extreme}\vert {H}_0\right),$$

we can now see that the *H*_0_ which the *P*-value and PDC can actually test must be

$$H_0:\left\{\left({\mu}_1={\mu}_2\right)\ \mathrm{and}\ \right[\left({\overline{x}}_1\ne {\overline{x}}_2)\ \mathrm{due}\ \mathrm{to}\ \mathrm{chance}\ \mathrm{alone}\right\}.$$

In other words, it is the hypothesis that the finding in the sample groups is due to chance or random variation alone and does not reflect a difference in the underlying population.

### Rejecting (*μ*_1_ = *μ*_2_)

Textbooks often claim that we can use NHST to reject (*μ*_1_ = *μ*_2_). However, this is not logically possible with the minimum axiom set NHST. To demonstrate this, we will need to transform (*μ*_1_ = *μ*_2_) to a logically equivalent proposition and use propositional calculus. The proposition (*μ*_1_ = *μ*_2_) is a proposition about the equality of the population means, but states nothing about the sample group means $(\overline{x})$. Using a truth table (Table 1), we can rewrite (*μ*_1_ = *μ*_2_) in a logically equivalent way such that the sample group means do appear in the proposition but without any claim being made about them.^2^ Note that *P*($\overline{x}$_1_ = $\overline{x}$_2_) =0, so any proposition containing ($\overline{x}$_1_ = $\overline{x}$_2_) can be eliminated from the analysis.

**Table 1:** Truth table for (*μ*_1_ = *μ*_2_) and its logical equivalent

*μ*_1_ = *μ*_2_	($\overline{x}$_1_ ≠ $\overline{x}$_2_) due to chance alone	($\overline{x}$_1_ ≠ $\overline{x}$_2_) not due to chance alone	{(*μ*_1_ = *μ*_2_) ∧ [($\overline{x}$_1_ ≠ $\overline{x}$_2_) due to chance alone]}	{(*μ*_1_ = *μ*_2_) ∧ [($\overline{x}$_1_ ≠ $\overline{x}$_2_) not due to chance alone]}	({(*μ*_1_ = *μ*_2_) ∧ [($\overline{x}$_1_ ≠ $\overline{x}$_2_) due to chance alone]} ∨ {(*μ*_1_ = *μ*_2_) ∧ [($\overline{x}$_1_ ≠ $\overline{x}$_2_) not due to chance alone]} )
T	T	F	T	F	T
T	F	T	F	T	T
F	T	F	F	F	F
F	F	T	F	F	F

From Table 1, (*μ*_1_ = *μ*_2_) ≡

1 $$\left(\left\{\left({\mu}_1={\mu}_2\right)\ \wedge\ \left[\left({\overline{x}}_1\ne {\overline{x}}_2\right)\ \mathrm{due}\ \mathrm{to}\ \mathrm{chance}\ \mathrm{alone}\right]\right\}\ \lor\ \left\{\left({\mu}_1={\mu}_2\right)\ \wedge\ \left[\left({\overline{x}}_1\ne {\overline{x}}_2\right)\ \mathrm{not}\ \mathrm{due}\ \mathrm{to}\ \mathrm{chance}\ \mathrm{alone}\right]\right\}\right).$$

Logical equivalence is established because whenever (*μ*_1_ = *μ*_2_) is true, 1 is true too, and whenever (*μ*_1_ = *μ*_2_) is false, 1 is also false. This transformation now allows us to see why eliminating the testable *H*_0_ does not logically imply the elimination of (*μ*_1_ = *μ*_2_). Let the first disjunct of 1 be called C, and the second disjunct E. Thus, 1 becomes the disjunction C v E. We recognise C as the testable *H*_0_. The PDC can assess C, and so it may be possible to reject C depending on the *P*-value. However, the PDC cannot assess E. So even if we do reject C, we cannot reject E, and therefore we cannot reject the whole proposition C v E. Since 1 is logically equivalent to (*μ*_1_ = *μ*_2_), we see that we cannot reject (*μ*_1_ = *μ*_2_) using the minimum axiom set NHST. In other words, (*μ*_1_ = *μ*_2_) is not rejected when we reject the testable *H*_0_: {(*μ*_1_ = *μ*_2_) ∧ [(${\overline{x}}_1$ ≠ ${\overline{x}}_2$) due to chance alone]}. To reject (*μ*_1_ = *μ*_2_), a further premise will need to be added, namely ¬{(*μ*_1_ = *μ*_2_) ∧ [(${\overline{x}}_1$ ≠ ${\overline{x}}_2$) not due to chance alone]}.

### The real *H*_A_

We take it as axiomatic that *H*_0_ and *H*_A_ are mutually exclusive: the hypotheses should not overlap in the sample space. An issue identified in the introduction was whether the hypothesis pair should also be exhaustive. There are serious consequences when the pair are made into a false dichotomy. An obvious criticism is that other possibilities are simply ignored. Furthermore, it opens a Pandora’s box of candidates for *H*_A_. Frequently the research or test hypothesis (here *H*_T_) is proposed as *H*_A_. This is the proposition that there is a difference in the population due to the study intervention or treatment and the finding in the sample groups is due to this difference alone. In symbols

$${H}_T:\{\left({\mu}_1\ne {\mu}_2\right)\ \wedge\ \left[\left({\overline{x}}_1\ne {\overline{x}}_2\right)\ \mathrm{due}\ \mathrm{to}\ \left({\mu}_1\ne {\mu}_2\right)\ \mathrm{alone}\right]\}.$$

However, if false dichotomies are allowed, what is to prevent other hypotheses being proposed as *H*_A_? Such as the hypothesis that bias or confounding produced the results, or some other hypothesis, or even combinations of hypotheses given that they are all independent propositions. In a false dichotomy the selection of *H*_A_ is subject to prejudice.

The above problems are avoided by forming an exhaustive hypothesis pair. To avoid logical errors of negation, it is critical to note that *H*_A_ must be the negation of the entire proposition represented by *H*_0_, not just a negation of part of *H*_0_. So *H*_A_ must be ¬*H*_0_ and the real *H*_A_: ¬{(*μ*_1_ = *μ*_2_) and [(${\overline{x}}_1$ ≠ ${\overline{x}}_2$) due to chance alone]}. Therefore, the only justifiable exhaustive hypothesis pair is

$${H}_0:\left\{\left({\mu}_1={\mu}_2\right)\ \mathrm{and}\ \left[\left({\overline{x}}_1\ne {\overline{x}}_2\right)\ \mathrm{due}\ \mathrm{to}\ \mathrm{chance}\ \mathrm{alone}\right]\right\},$$

$${H}_A:\neg \left\{\left({\mu}_1={\mu}_2\right)\ \mathrm{and}\ \left[\left({\overline{x}}_1\ne {\overline{x}}_2\right)\ \mathrm{due}\ \mathrm{to}\ \mathrm{chance}\ \mathrm{alone}\right]\right\}.$$

### The relationship between *H*_A_ and *H*_T_

*H*_A_ is a more complex proposition than *H*_T_. Once again, we can transform *H*_A_ into a logically equivalent proposition which has *H*_T_ as a component. Let *H*_A_ be represented by ¬(G ∧ J), where G is “*μ*_1_ = *μ*_2_”, and J is “ $(\overline{x}$_1_ ≠ $\overline{x}$_2_) due to chance alone.” The truth table for ¬(G ∧ J) is shown in Table 2.

**Table 2:** Truth table for ¬(G ∧ J)

G	¬G	J	¬J	G ∧ J	¬(G ∧ J)
T	F	T	F	T	F
**T**	F	F	**T**	F	**T**
F	**T**	**T**	F	F	**T**
F	**T**	F	**T**	F	**T**

Table 2 shows that ¬(G ∧ J) is true (bold **T** in last column) when G and ¬J are true (the second row), or ¬G and J are true (the third row), or ¬G and ¬J are true (the last row). This allows us to formulate a disjunction logically equivalent to ¬(G ∧ J). Thus ¬(G ∧ J) ≡ (G ∧ ¬J) ∨ (¬G ∧ J) ∨ (¬G ∧ ¬J). Now ¬J ≡ {($\overline{x}$_1_ = $\overline{x}$_2_) ∨ [($\overline{x}$_1_ ≠ $\overline{x}$_2_) not due to chance alone]}. However, as stated previously, we can eliminate ($\overline{x}$_1_ = $\overline{x}$_2_) making ¬J ≡ [($\overline{x}$_1_ ≠ $\overline{x}$_2_) not due to chance alone]. Substituting back, *H*_A_ ≡

$$(\left\{\left({\mu}_1={\mu}_2\right)\ \wedge\ \left[\left({\overline{x}}_1\ne {\overline{x}}_2\right)\ \mathrm{not}\ \mathrm{due}\ \mathrm{to}\ \mathrm{chance}\ \mathrm{alone}\right]\right\}\ \lor\ \left\{\left({\mu}_1\ne {\mu}_2\right)\ \wedge\ \left[\left({\overline{x}}_1\ne {\overline{x}}_2\right)\ \mathrm{due}\ \mathrm{to}\ \mathrm{chance}\ \mathrm{alone}\right]\right\}\ \lor\ \left\{\left({\mu}_1\ne {\mu}_2\right)\ \wedge\ \left[\left({\overline{x}}_1\ne {\overline{x}}_2\right)\ \mathrm{not}\ \mathrm{due}\ \mathrm{to}\ \mathrm{chance}\ \mathrm{alone}\right]\right\}).$$

Furthermore, the second disjunct is a contradiction and can be eliminated giving

2 $${\it{H}}_{\mathrm{A}}:\left\{\left({\mu}_1={\mu}_2\right)\ \wedge\ \left[\left({\overline{x}}_1\ne {\overline{x}}_2\right)\ \mathrm{not}\ \mathrm{due}\ \mathrm{to}\ \mathrm{chance}\ \mathrm{alone}\right]\right\}\ \lor\ \left\{\left({\mu}_1\ne {\mu}_2\right)\ \wedge\ \left[\left({\overline{x}}_1\ne {\overline{x}}_2\right)\ \mathrm{not}\ \mathrm{due}\ \mathrm{to}\ \mathrm{chance}\ \mathrm{alone}\right]\right\}.$$

Where does *H*_T_ lie in 2? *H*_T_ is contained within the last disjunct of 2, {(*μ*_1_ ≠ *μ*_2_) ∧ [($\overline{x}$_1_ ≠ $\overline{x}$_2_) not due to chance alone]}. The latter disjunct expresses the proposition that there is a difference found in the population and also that the sample group difference is not due to chance alone, but instead is due to some other alternative. The other alternatives include the test intervention or bias or some other unknown or even a combination of these given that the alternatives are independent hypotheses. Taking this into account we can rewrite 2 such that *H*_A_ ≡

3 $$\left\{\left({\mu}_1={\mu}_2\right)\ \wedge\ \left[\left({\overline{x}}_1\ne {\overline{x}}_2\right)\ \mathrm{not}\ \mathrm{due}\ \mathrm{to}\ \mathrm{chance}\ \mathrm{alone}\right]\right\}\ \lor\ \left\{\left({\mu}_1\ne {\mu}_2\right)\ \wedge\ \left[\left({\overline{x}}_1\ne {\overline{x}}_2\right)\ \mathrm{not}\ \mathrm{due}\ \mathrm{to}\ \left({\mu}_1\ne {\mu}_2\right)\ \mathrm{alone}\right]\right\}\ \lor\ \left\{\left({\boldsymbol{\mu}}_{\mathbf{1}}\mathbf{\ne}{\boldsymbol{\mu}}_{\mathbf{2}}\right)\ \boldsymbol{\wedge}\ \left[\left({\overline{\boldsymbol{x}}}_{\mathbf{1}}\mathbf{\ne}{\overline{\boldsymbol{x}}}_{\mathbf{2}}\right)\ \mathbf{due}\ \mathbf{to}\ \left({\boldsymbol{\mu}}_{\mathbf{1}}\mathbf{\ne}{\boldsymbol{\mu}}_{\mathbf{2}}\right)\ \mathbf{alone}\right]\right\}.$$

The last disjunct of 3 is *H*_T_ (in bold), indicating that *H*_T_ is just one sub-hypothesis of *H*_A_.

Finally, the answer to the question “What do we accept when we reject *H*_0_?” is: we accept the real *H*_A_ or its logical equivalent (3). Therefore, a statistically significant finding, expressed in these common terms, should be interpreted as meaning that the data is not due to chance alone. Statistical significance is not a licence to accept *H*_T_.

### The effect of further premises on the minimum axiom set NHST

It is only by adding premises to NHST that we can conclude anything other than the real *H*_A_. The danger with this strategy is that of partially assuming what is being proved. Table 3 presents examples of premises that if added to NHST would rig different conclusions.

**Table 3:** Adding premises to NHST to conclude *H*_T_. Comparison of group means is used as an example. *H*_T_ (in bold) is defined in the text

Perform NHST: if ***P***-value ≥ α, then fail to reject ***H***_**0.**_ If ***P***-value < α, ***H***_**0**_ is rejected and conclude ***H***_**A**_: ¬{(***μ***_**1**_ = ***μ***_**2**_) ∧ [($\overline{\boldsymbol{x}}$_**1**_ ≠ $\overline{\boldsymbol{x}}$_**2**_) due to chance alone]}			
Further steps	Aim to conclude ***H***_**T**_	Assume “there is no bias”	Aim to conclude ***H***_**B**_
**Additional premises**	(1) ¬{(*μ*_1_ = *μ*_2_) ∧ [($\overline{x}$_1_ ≠ $\overline{x}$_2_) not due to chance alone]} (2) ¬{(*μ*_1_ ≠ *μ*_2_) ∧ [($\overline{x}$_1_ ≠ $\overline{x}$_2_) not due to (*μ*_1_ ≠ *μ*_2_) alone nor chance alone]}	(1) ¬{(*μ*_1_ = *μ*_2_) ∧ [($\overline{x}$_1_ ≠ $\overline{x}$_2_) due to bias]}	(1) ¬ {(*μ*_1_ = *μ*_2_) ∧ [($\overline{x}$_1_ ≠ $\overline{x}$_2_) not due to bias alone nor chance alone]} (2) (*μ*_1_ = *μ*_2_)
**Reasoning**	*H*_A_: ¬{(*μ*_1_ = *μ*_2_) ∧ [($\overline{x}$_1_ ≠ $\overline{x}$_2_) due to chance alone]} ≡ *H*_A_: ({(*μ*_1_ = *μ*_2_) ∧ [($\overline{x}$_1_ ≠ $\overline{x}$_2_) not due to chance alone]} ∨ {(*μ*_1_ ≠ *μ*_2_) ∧ [($\overline{x}$_1_ ≠ $\overline{x}$_2_) not due to (*μ*_1_ ≠ *μ*_2_) alone nor chance alone]} ∨ **{(*μ***_**1**_ **≠** ***μ***_**2**_**) ∧ [(**$\overline{\boldsymbol{x}}$_**1**_ **≠** $\overline{\boldsymbol{x}}$_**2**_**) due to (*μ***_**1**_ **≠** ***μ***_**2**_**) alone]}**). Use 2 steps of disjunction elimination with (1) and (2)	*H*_A_: ¬{(*μ*_1_ = *μ*_2_) ∧ [($\overline{x}$_1_ ≠ $\overline{x}$_2_) due to chance alone]} ≡ *H*_A_: ({(*μ*_1_ = *μ*_2_) ∧ [($\overline{x}$_1_ ≠ $\overline{x}$_2_) not due to bias nor chance alone]} ∨ {(*μ*_1_ = *μ*_2_) ∧ [($\overline{x}$_1_ ≠ $\overline{x}$_2_) due to bias]} ∨ **{(*μ***_**1**_ **≠** ***μ***_**2**_**) ∧ [(**$\overline{\boldsymbol{x}}$_**1**_ **≠** $\overline{\boldsymbol{x}}$_**2**_**) due to (*μ***_**1**_ **≠** ***μ***_**2**_**) alone]}** ∨ {(*μ*_1_ ≠ *μ*_2_) ∧ [($\overline{x}$_1_ ≠ $\overline{x}$_2_) not due to (*μ*_1_ ≠ *μ*_2_) alone]}). Use disjunction elimination with (1)	*H*_A_: ¬{(*μ*_1_ = *μ*_2_) ∧ [($\overline{x}$_1_ ≠ $\overline{x}$_2_) due to chance alone]} ≡ *H*_A_: ({(*μ*_1_ = *μ*_2_) ∧ [($\overline{x}$_1_ ≠ $\overline{x}$_2_) due to bias alone]} ∨ {(*μ*_1_ = *μ*_2_) ∧ [($\overline{x}$_1_ ≠ $\overline{x}$_2_) not due to bias alone nor chance alone]} ∨ (*μ*_1_ ≠ *μ*_2_)). Use 2 steps of disjunction elimination with (1) and (2)
**Conclusion**	Therefore **{(*μ***_**1**_ **≠** ***μ***_**2**_**) ∧ [(**$\overline{\boldsymbol{x}}$_**1**_ **≠** $\overline{\boldsymbol{x}}$_**2**_**) due to (*μ***_**1**_ **≠** ***μ***_**2**_**) alone]}, i.e.,** ***H***_**T**_	Therefore ({(*μ*_1_ = *μ*_2_) ∧ [($\overline{x}$_1_ ≠ $\overline{x}$_2_) not due to bias nor chance alone]} ∨ {(*μ*_1_ ≠ *μ*_2_) ∧ [($\overline{x}$_1_ ≠ $\overline{x}$_2_) not due to (*μ*_1_ ≠ *μ*_2_) alone]} ∨ **{(*μ***_**1**_ **≠** ***μ***_**2**_**) ∧ [(**$\overline{\boldsymbol{x}}$_**1**_ **≠** $\overline{\boldsymbol{x}}$_**2**_**) due to (*μ***_**1**_ **≠** ***μ***_**2**_**) alone]}**)	Therefore {(*μ*_1_ = *μ*_2_) ∧ [($\overline{x}$_1_ ≠ $\overline{x}$_2_) due to bias alone]}, i.e., *H*_B_

Some texts claim that all that is needed to conclude *H*_T_ when *H*_0_ is rejected is the assumption that there is no bias [35, 47]. However, Table 3 illustrates exactly which premises are needed in order to conclude *H*_T_. Apart from assuming no bias, it is also necessary to assume there are no combination hypotheses in which chance plays a role. A corollary is that if NHST could lead us to conclude *H*_T_ of its own accord, no further premises would be required. What would the conclusion be if indeed we only assumed that there was no bias? The middle column of Table 3 shows the conclusion. In a model which stipulates that the possible causes of the sample group difference are chance, bias or the intervention (or combinations thereof), the conclusion would be

$${\boldsymbol\{{\boldsymbol({\boldsymbol{\mu}}_{\mathbf{1}}\ {\boldsymbol\ne}\ {\boldsymbol{\mu}}_{\mathbf{2}}\boldsymbol)}\ \boldsymbol\wedge\ {\boldsymbol[{\boldsymbol({\boldsymbol{\overline{\boldsymbol{x}}}_{\mathbf{1}}}\ \mathbf{\boldsymbol\ne}\ {\boldsymbol{\overline{\boldsymbol{x}}}_{\mathbf{2}}}\boldsymbol)}\ \mathbf{due}\ \mathbf{to}\ {\boldsymbol({\boldsymbol{\mu}}_{\mathbf{1}}\ {\boldsymbol\ne}\ {\boldsymbol{\mu}}_{\mathbf{2}}\boldsymbol)}\ \mathbf{alone}\boldsymbol]}\boldsymbol\}}\ \lor\ \left\{\right[\left({\mu}_1\ne {\mu}_2\right)\ \wedge\ \left[\left({\overline{x}}_1\ne {\overline{x}}_2\right)\ \mathrm{due}\ \mathrm{to}\ \left({\mu}_1\ne {\mu}_2\right)\ \mathrm{and}\ \mathrm{chance}\right]\}.$$

The first disjunct in bold is *H*_T_, showing that the conclusion is more complex than *H*_T_ alone. The last column demonstrates that a different package of additional premises can be tailored to reach a different conclusion such as the hypothesis that bias produced the results, here represented as *H*_B_: {(*μ*_1_ = *μ*_2_) ∧ [($\overline{x}$_1_ ≠ $\overline{x}$_2_) due to bias alone]}. Similar to arithmetic, the process in Table 3 is commutative. The same results are achieved if we were to make the assumptions first and then do the NHST or vice versa ― the order does not matter.

### Application to other statistical problems

So far we have focused on the comparison of sample group means. However, with appropriate changes in vocabulary we can define the real *H*_0_ and *H*_A_ for other scenarios ― mutatis mutandis*,* as they say. As illustrations, *H*_0_ and *H*_A_ in general form, for the comparison of sample group proportions, and for correlation are presented in Table 4.

**Table 4:** *H*_0_ and *H*_A_ for common scenarios. *H*_A_ has also been transformed into its logical equivalent to identify *H*_T_ (in bold)

Scenario	General Form	Comparing proportions (Chi-squared test)	Correlation
***H***_**0**_ **and** ***H***_**A**_	*H*_0_: there is no finding in the population and the finding in the sample group is due to chance alone *H*_A_: it is not the case that *H*_0_, therefore *H*_A_: it is not the case that (there is no finding in the population and the finding in the sample group is due to chance alone) *≡* [(there is no finding in the population and the finding in the sample group is not due to chance alone) or (there is a finding in the population and the finding in the sample group is not due to the population finding alone) or **(there is a finding in the population and the finding in the sample group is due to the population finding alone)]**	*H*_0_: (p̂_1_ = p̂_2_) ∧ [(p_1_ ≠ p_2_) due to chance alone] *H*_A_: ¬*H*_0_, therefore *H*_A_: ¬{(p̂_1_ = p̂_2_) ∧ [(p_1_ ≠ p_2_) due to chance alone]} ≡ *H*_A:_ ({(p̂_1_ = p̂_2_) ∧ [(p_1_ ≠ p_2_) not due to chance alone]} ∨ {(p̂_1_ ≠ p̂_2_) ∧ [(p_1_ ≠ p_2_) not due to (p̂_1_ ≠ p̂_2_) alone]} ∨ **{(p̂**_**1**_ **≠ p̂**_**2**_**) ∧ [(p**_**1**_ **≠ p**_**2**_**) due to (p̂**_**1**_ **≠ p̂**_**2**_**) alone]})**	*H*_0_: (*ρ* = 0) ∧ (*r* ≠ 0 due to chance alone) *H*_A_: ¬*H*_0_, therefore *H*_A_: ¬[(*ρ* = 0) ∧ (*r* ≠ 0 due to chance alone)] ≡ *H*_A:_ ([(*ρ* = 0) ∧ (*r* ≠ 0 not due to chance alone)] ∨ {(*ρ* ≠ 0) ∧ [*r* ≠ 0 not due to (*ρ* ≠ 0) alone]} ∨ **{(*ρ*** **≠ 0) ʌ [*r*** **≠ 0 due to (*ρ*** **≠ 0) alone]}**)

### Failure to reject *H*_0_

What are we to conclude if we fail to reject *H*_0_? The axiom of NHST states that we reject *H*_0_ if *P*-value < α. This does not logically imply that if *P*-value ≥ α we must accept *H*_0_ ― the axiom and the claim about accepting *H*_0_ are logically distinct ideas. So if *P*-value ≥ α, we should merely state we have failed to reject *H*_0_ rather than we accept *H*_0_.

### Power (1-*β*), type I (*α*) and type II (*β*) errors

Textbooks which express NHST in terms of the research hypothesis also tend to carry this over to descriptions of Type I and II errors, as well as power calculations. However, this is fraught with error as can be seen when we apply the real definitions of *H*_0_ and *H*_A_. Type I error is the probability of eliminating *H*_0_, and accepting *H*_A_, when in fact *H*_0_ is true. Using the real definitions of *H*_0_ and *H*_A_ gives us type I error:

$$P\left(\mathrm{rejecting}\ \left\{\left({\mu}_1={\mu}_2\right)\ \mathrm{and}\ \left[\left({\overline{x}}_1\ne {\overline{x}}_2\right)\ \mathrm{due}\ \mathrm{to}\ \mathrm{chance}\ \mathrm{alone}\right]\right\}\vert \left\{\left({\mu}_1={\mu}_2\right)\ \mathrm{and}\ \left[\left({\overline{x}}_1\ne {\overline{x}}_2\right)\ \mathrm{due}\ \mathrm{to}\ \mathrm{chance}\ \mathrm{alone}\right]\right\}\right).$$

Importantly, type I error is not the probability of accepting *H*_T_ when *H*_0_ is true. Since *H*_A_ is a disjunction, there are multiple propositions that can make it true, with *H*_T_ being just one of these. So *P*(*H*_A_) > *P*(*H*_T_) and *P*(mistakenly accepting *H*_T_) > *P*(mistakenly accepting *H*_A_). The conflation of *H*_T_ with *H*_A_ results in underestimating the probability of mistakenly accepting *H*_T_.

Similarly for type II error which is the probability of not rejecting *H*_0_, and not accepting *H*_A_, when *H*_0_ is false and should have been rejected. Namely,

$$P\left(\mathrm{not}\ \mathrm{rejecting}\ \left\{\left({\mu}_1={\mu}_2\right)\ \mathrm{and}\ \left[\left({\overline{x}}_1\ne {\overline{x}}_2\right)\ \mathrm{due}\ \mathrm{to}\ \mathrm{chance}\ \mathrm{alone}\right]\right\}\vert \mathrm{it}\ \mathrm{is}\ \mathrm{not}\ \mathrm{the}\ \mathrm{case}\ \mathrm{that}\ \left\{\left({\mu}_1={\mu}_2\right)\ \mathrm{and}\ \left[\left(\ {\overline{x}}_1\ne {\overline{x}}_2\right)\ \mathrm{due}\ \mathrm{to}\ \mathrm{chance}\ \mathrm{alone}\right]\right\}\right).$$

Type II error is not the probability of not accepting *H*_T_ when *H*_0_ is false. A low probability of not accepting *H*_A_ does not logically imply a low probability of not accepting *H*_T_. *P*(not accepting *H*_T_) > *P*(not accepting *H*_A_) because more propositions need to be rejected in order to accept *H*_T_. The conflation of *H*_T_ with *H*_A_ results in underestimating the probability of not accepting *H*_T_ when *H*_0_ is false.

Power (1- β) refers to the probability of rejecting *H*_0_ and accepting *H*_A_ given *H*_0_ is false. Specifically, power is

$$P\left(\mathrm{rejecting}\ \left\{\left({\mu}_1={\mu}_2\right)\ \mathrm{and}\ \ \left[\left({\overline{x}}_1\ne {\overline{x}}_2\right)\ \mathrm{due}\ \mathrm{to}\ \mathrm{chance}\ \mathrm{alone}\right]\right\}\vert \mathrm{it}\ \mathrm{is}\ \mathrm{not}\ \mathrm{the}\ \mathrm{case}\ \mathrm{that}\ \left\{\left({\mu}_1={\mu}_2\right)\ \mathrm{and}\ \ \left[\left({\overline{x}}_1\ne {\overline{x}}_2\right)\ \mathrm{due}\ \mathrm{to}\ \mathrm{chance}\ \mathrm{alone}\right]\right\}\right).$$

However, it does not refer to *P*(accepting *H*_T_│*H*_T_). The power to conclude *H*_T_ < the power to conclude *H*_A_. The conflation of *H*_T_ with *H*_A_ results in overestimating the power to conclude *H*_T_ because *H*_T_ is just one part of *H*_A_.

## Discussion

NHST has been well described in terms of statistical models. However, it is also commonly presented in terms of group comparisons and with reference to the research hypothesis. Despite this being a popular interpretation, there is currently no standardised approach. The variation in definitions of *H*_0_ and *H*_A_, how they should be paired and conclusions that can be drawn by eliminating *H*_0_ motivated this new logical analysis. Looking at the conditions of the *P*-value we can see that there can be only one testable *H*_0_. Presenting *H*_0_ and *H*_A_ as a false dichotomy is common but unjustifiable. Combining these two ideas entails that *H*_A_ is ¬*H*_0_. Texts should acknowledge this and also make transparent any premises added in order to reach a conclusion other than ¬*H*_0_ when *H*_0_ is rejected.

It may be thought that using the estimation or CI method can avoid the problems of expressing NHST in these terms. However, this is not true if the estimation method is used as a de facto NHST. The estimation method can be used as a NHST because the CI is mathematically related to the *α*-level and the *P*-value such that if the CI does not cross zero (or 1 for ratios), we can claim statistical significance. In the context of using CI as a NHST, the conclusions of the present paper are relevant. Consequently, when using the CI method, the correct interpretation of statistical significance would be to accept the real *H*_A_ and not claim that *H*_T_ is true. Of course, there are other appealing features of the CI method and the present discussion is limited only to its use as a significance test.

A limitation of the present paper is that we have not questioned the axiom of NHST that we reject *H*_0_ if the *P*-value < α. An analysis of this axiom deserves a paper in its own right which discusses inductive logic and defines the conditions under which the axiom is reliable. The issue in the present paper has been solely that if we are to use NHST as it is commonly presented it should at least be with justifiable definitions of *H*_0_ and *H*_A_, transparent assumptions and valid deductions from the given premises.

## Conclusions

NHST is commonly expressed in terms of differences between groups and with reference to the research hypothesis. Within this framework, logical analysis reveals that the minimum axiom set NHST (for comparing sample means) is as follows:

- *H*_0_: {(*μ*_1_ = *μ*_2_) and [($\overline{x}$_1_ ≠ $\overline{x}$_2_) due to chance alone]},
- *H*_A_: ¬{(*μ*_1_ = *μ*_2_) and [($\overline{x}$_1_ ≠ $\overline{x}$_2_) due to chance alone]}.
- If *P*-value ≥ α, then fail to reject *H*_0._
- If *P*-value < α, reject *H*_0_ and conclude *H*_A._

At best, it can be concluded that if *H*_0_ is rejected, the data were not due to chance alone. Texts should also be transparent about which assumptions have been added to rig a conclusion such as *H*_T_. Care should also be exerted to avoid misinterpreting type I and II errors, as well as power, in terms of the research hypothesis.

## Data Availability

All data generated or analysed during this study are included in this published article.

## Footnotes

1 “NHST” is probably the most widely used abbreviation for the various names applied to hypothesis and significance tests 1. Nickerson RS. Null hypothesis significance testing: a review of an old and continuing controversy. *Psychol Methods* 2000; 5: 241–301. 2000/08/11. DOI: 10.1037/1082-989x.5.2.241.

2 Truth tables analyse the truth of complex propositions based on giving truth values of true (T) or false (F) to its elemental components. When propositions are subject to logical analysis here, we shall use the symbols of propositional calculus: “∧” for “and”; “∨” for “or”; and “¬” for “not” used to express negation. “¬X” means “It is not the case that X.” “≡” means “is equivalent to” such that “X ≡ Y” means “proposition X is equivalent to proposition Y.”
